# Postoperative Corneal and Surgically Induced Astigmatism following Superior Approach Manual Small Incision Cataract Surgery in Patients with Preoperative Against-the-Rule Astigmatism

**DOI:** 10.1155/2016/9489036

**Published:** 2016-12-28

**Authors:** Edmund Arthur, Ahmed Abdul Sadik, David Ben Kumah, Eugene Appenteng Osae, Felix Agyemang Mireku, Frank Yeboah Asiedu, Reynolds Kwame Ablordeppey

**Affiliations:** ^1^Department of Optometry and Visual Science, Kwame Nkrumah University of Science and Technology, Kumasi, Ghana; ^2^Ruebsam Eye Clinic, St. Dominic's Hospital, P.O. Box 59, Akwatia, Ghana

## Abstract

The aim of the study was to report postoperative corneal and surgically induced astigmatism (SIA) in patients with preoperative against-the-rule (ATR) astigmatism who underwent superior approach manual small incision cataract surgery (MSICS). 58 eyes of 58 cataract patients with preoperative ATR astigmatism were involved in this study. All patients had operable cataracts and underwent superior approach MSICS. Keratometric (*K*) readings were taken prior to surgery and at 12 weeks after surgery. Centroid values of SIA, preoperative astigmatism, and postoperative astigmatism were calculated using Cartesian coordinates based analysis. Wilcoxon signed rank test was used to compute statistical significance between mean preoperative and postoperative corneal astigmatism. Cohen's *d* was used as effect size measure. Centroid values of 1.42 D × 179, 2.48 D × 0, and 1.07 D × 1 were recorded, respectively, for preoperative astigmatism, postoperative astigmatism, and SIA. Wilcoxon signed rank test indicated that mean ± SD postoperative corneal astigmatism (2.80 ± 1.40 D) was statistically significantly greater than preoperative corneal astigmatism (1.49 ± 1.34 D), *Z* = −6.263, *p* < 0.0001. A high Cohen's *d* of 1.32 was found. Our results suggest statistical and clinically significant greater postoperative corneal astigmatism than preoperative corneal astigmatism for ATR astigmatism cataract patients who underwent superior approach MSICS.

## 1. Introduction

Cataract poses both a significant socioeconomic burden and a public health concern as it is the leading cause of blindness worldwide [1] and a major cause of visual disability throughout the African continent [2–5]. The current treatment for cataract is surgery [6, 7] and while phacoemulsification remains the more advanced and technically superior method of cataract surgery, manual small incision cataract surgery (MSICS) is the most popular surgical management option for cataracts in developing countries [8–11]. This is mainly because of the low cost, short surgical time, reduced dependence on technology, and equivalent visual outcome to phacoemulsification [8–11].

The location, size, and shape of incisions used in MSICS influence postoperative surgically induced astigmatism (SIA) [12–14]. Temporal approach has been reported to result in smaller SIA than superior approach [12]. Small incisions (6 mm) induced the smallest SIA when compared with medium (6.5 mm) and large (7 mm) incisions [13]. The chevron shaped incision has also been reported to give minimal SIA when compared with straight and frown incisions [14]. Corneal or keratometric SIA is the vector difference between the preoperative corneal or keratometric astigmatism and the postoperative astigmatism [15].

With reference to the location of the incision, placing the incision on the steeper corneal meridian based on the preoperative keratometric (*K*) reading has been recommended [16]. The idea is that because of the one-to-one coupling from corneal incisions, there is flattening of the corneal curvature in the meridian on which the incision is placed, with a corresponding steepening to the same degree of the orthogonal meridian [17]. Thus, there will be a reduction in the corneal power of the steeper meridian when an incision is placed on that meridian, with a corresponding steepening to the same degree of the flat orthogonal meridian. The difference in corneal powers between the flattened steeper meridian (meridian on which the incision was placed) and the steepened flatter meridian will then be reduced postoperatively leading to minimal postoperative corneal astigmatism.

With increasing age, the horizontal corneal meridian becomes more curved than the vertical meridian leading to or increasing existing against-the-rule (ATR) astigmatism [18]. Thus, there is an ATR shift in astigmatism with age. Placing an incision on the vertical meridian (superior approach) for a cataract patient with preoperative ATR astigmatism may cause further flattening of the already flatter vertical meridian and a corresponding steepening to the same degree of the already steeper horizontal meridian leading to high postoperative corneal astigmatism. With senile cataract being the most common type of cataract in developing countries [19] and since there is an ATR shift in astigmatism with age [18], most cataract patients in developing countries may have preoperative ATR astigmatism. Hence, the choice of the location of incision for these groups of patients is important.

Even though other studies have reported on the postoperative corneal astigmatism and SIA accompanying the size, shape, and location of incisions performed in MSICS in developing countries, none of these studies concentrated on a cohort of ATR astigmatism cataract patients who underwent superior approach MSICS [12–14, 20]. We herein present a report on the postoperative corneal astigmatism and SIA for ATR astigmatism cataract patients who underwent superior approach MSICS in a developing country. The outcome of the study may help improve postoperative visual acuity (VA) and reduce the spectacle burden on the low-income patient.

## 2. Materials and Methods

### 2.1. Subjects

A prospective study involving 58 subjects diagnosed with cataract in a secondary eye unit was carried out. The subjects were comprised of 39 females and 19 males. A total of 28 and 30 right and left eyes, respectively, were involved in the study. Only subjects with preoperative ATR astigmatism who had operable cataracts with no history of corneal diseases were included in the study. Subjects who developed any postoperative complications were excluded. Preoperative ATR astigmatism was defined as having an axis of corneal astigmatism of 90 ± 20 degrees (minus cylinder form) or 180 ± 20 degrees (plus cylinder form) based on the preoperative keratometry (*K*) readings [21].

### 2.2. Ethical Consideration

Informed consent was obtained from all subjects after the procedure and aim of the study were described to them. Subjects were also told that they could withdraw from the study at any time. The study was approved by the Ethics and Research committees of St. Dominic's Hospital and the Department of Optometry and Visual Science, Kwame Nkrumah University of Science and Technology, and was carried out in accordance with the tenets of the Declaration of Helsinki.

### 2.3. Preoperative and Postoperative Measurements

*K* readings were measured prior to surgery and at 12 weeks after surgery. *K* readings were measured using a dual function Topcon autorefractor and keratometer (Topcon KR 8900). They were taken at 12 weeks postoperatively because SIA would have stabilized by that time [22]. Even though subjects came for routine postsurgical management, the preoperative and the 12-week postoperative keratometric measurements were the variables of interest. Application of daily ciprofloxacin drops one week prior to surgery was also done. The decision to do MSICS was made during the preoperative assessment. Biometry was obtained through contact method (Tomey AL 100) and intraocular lens (IOL) power was calculated using the Haigis optimized formula.

### 2.4. Surgical Procedure

On the day of surgery, the pupil was dilated with 0.8% tropicamide and 5% phenylephrine drops. The surgery was performed under retrobulbar anesthesia. All of the surgeries were performed by one surgeon. The surgical procedure is similar to a previously described MSICS procedure [14]. After making a fornix based conjunctival flap, a 6 mm straight incision was made 2 mm away from the superior limbus (superior approach). A sterile disposable 2.8 mm crescent blade was used to create a self-sealing scleral corneal tunnel, extending into the clear cornea for 1 mm. A 3.2 mm keratome was used to enter the anterior chamber through the tunnel incision. Continuous curvilinear capsulorhexis was done with a 26 G cystostome through the main tunnel under viscoelastic cover.

Hydrodissection and delineations were then performed. The prolapsed lens was engaged in the scleral tunnel and delivered out using irrigating vectis. Irrigation and aspiration of any remaining cortical lens matter were done. A single PMMA IOL was implanted in the capsular bag and dialed. The self-sealing wound was then left without suturing after checking for any wound leakage.

### 2.5. Preoperative, Postoperative Corneal, and Surgically Induced Astigmatism (SIA)

The amount of SIA was calculated by the Cartesian coordinates based analysis using SIA calculator, version 2.1, by Dr. Saurabh Sawhney and Dr. Aashima Aggarwal [23, 24], and the ASSORT vector calculator in which the Alpins method of astigmatism analysis used in cataract surgery is programmed [15]. Preoperative and postoperative keratometric data were converted to a plus cylinder form in order to get preoperative and postoperative corneal astigmatism. This was essentially the difference in *K* readings between the two corneal meridians with the steeper axis taken as the axis of astigmatism. This transformation presupposes that the steeper and flatter meridians are at right angles as is the case in most people. *X* (horizontal) and *Y* (vertical) vectors were then generated from the preoperative and postoperative corneal astigmatic data for each subject using the formulae *x* = *a*cos⁡2*p* and *y* = *a*sin⁡2*p*, where *a* is the magnitude of astigmatism and *p* is the axis of the steeper meridian. Thus, we generated *X*_(pre)_, *Y*_(pre)_, *X*_(post)_, and *Y*_(post)_. To calculate the SIA for each subject, *X*_(pre)_ was subtracted from *X*_(post)_ to give *X*_SIA_. The same was done for *Y*_(pre)_ and *Y*_(post)_ to give *Y*_SIA_. The magnitude of the SIA was then found using the formula magnitude = (*X*_SIA_^2^ + *Y*_SIA_^2^)^1/2^ considering only the positive square root. The angle of the SIA was also found using the formula *θ* = 0.5 × arctan(*Y*_SIA_/*X*_SIA_).

The aggregate or the centroid SIA value for all subjects was calculated by finding the mean of all *X* values preoperatively and postoperatively [*X*_mean(pre)_, *X*_mean(post)_]. The same was done for the *Y* vectors [*Y*_mean(pre)_, *Y*_mean(post)_]. *X*_mean(pre)_ was then subtracted from *X*_mean(post)_ to get *X*_meanSIA_ and *Y*_mean(pre)_ was also subtracted from *Y*_mean(post)_ to get *Y*_meanSIA_. The magnitude of the centroid or aggregate SIA was then calculated as magnitude = (*X*_meanSIA_^2^ + *Y*_meanSIA_^2^)^1/2^ with the angle calculated as *θ* = 0.5 × arctan(*Y*_meanSIA_/*X*_meanSIA_). The centroid and angle of the preoperative corneal astigmatism of all subjects were calculated as magnitude = [*X*_mean(pre)_^2^ + *Y*_mean(pre)_^2^]^1/2^  and *θ* = 0.5 × arctan[*Y*_mean(pre)_/*X*_mean(pre)_], respectively. The centroid and angle of the postoperative corneal astigmatism of all subjects were also calculated as magnitude = [*X*_mean(post)_^2^ + *Y*_mean(post)_^2^]^1/2^ and *θ* = 0.5 × arctan[*Y*_mean(post)_/*X*_mean(post)_], respectively. The centroid values were presented as plus cylinders in diopters.

Coherence values were also shown to give the reliability of the centroid values. High coherence values posit high reliability and indicate that the centroid value is a true representation of the individual SIA and preoperative or postoperative corneal astigmatic values. Double-angle plots (DAP) were used to plot the various centroid values [23]. These plots have concentric circles demonstrating magnitude and axis of astigmatism from 0 to 180 degrees [23]. The 12 o'clock position of the circles shows 45°, 9 o'clock shows 90°, and 6 o'clock shows 135° axis of astigmatism [23].

### 2.6. Statistical Analysis

All statistical analyses were done using SPSS version 23.0 (IBM Corporation, Armonk, NY, USA). The Shapiro-Wilk test of normality was used to test the normal distribution of our preoperative and postoperative corneal astigmatism data. The Shapiro-Wilk test came out significant for both the preoperative and the postoperative corneal astigmatism data. Hence, the nonparametric Wilcoxon signed rank test was used to compare the means of the preoperative and postoperative corneal astigmatism values. For the purpose of comparing the mean preoperative and postoperative corneal astigmatism, only the magnitude of the corneal astigmatism was considered [15]. Cohen's *d* was used as the effect size measure to determine clinical significance and was calculated using GPower calculator 3.1 [25]. A Cohen's *d* value of 0.8 or greater was taken as high or clinically significant [26]. Microsoft Excel 2016 (Microsoft Corp., Redmond, WA, USA) was used for mean plots. The double-angle plots (DAP) were drawn with Sigma Plot 13.0 (Systat Software, San Jose, CA, USA). A *p* value < 0.05 was considered statistically significant. All values are presented as mean ± SD.

## 3. Results

### 3.1. Subjects' Demographics

58 eyes of 58 subjects were included in the study. All 58 subjects had preoperative and 12-week postoperative *K* readings measured. All subjects were ATR astigmatism cataract patients who underwent superior approach MSICS. The mean age of the subjects was 66.98 ± 10.92 years. In Table 1, the distribution of the age groups is illustrated; the age group 70–79 had the highest frequency.

### 3.2. Preoperative and Postoperative Corneal Astigmatism

**Figure 1:**
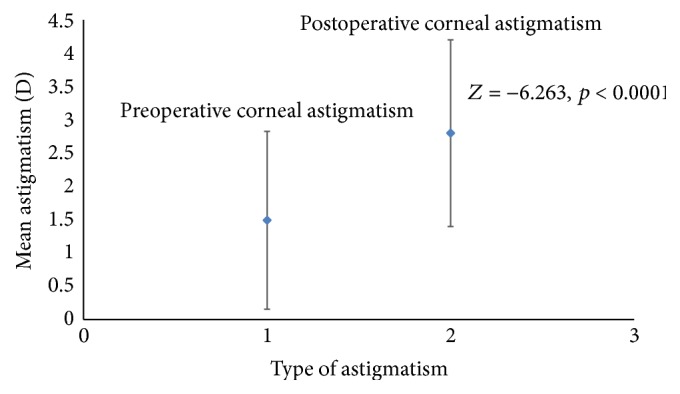
Mean plot of preoperative and postoperative corneal astigmatism with standard deviation error bars. A statistically significant difference is found between preoperative and postoperative corneal astigmatism (*p* < 0.0001).

The average preoperative and postoperative corneal astigmatism values were 1.49 ± 1.34 D and 2.80 ± 1.40 D, respectively. Wilcoxon signed rank test indicated that the mean postoperative corneal astigmatism was statistically significantly greater than the mean preoperative corneal astigmatism, *Z* = −6.263, *p* < 0.0001 (Figure 1). The effect size was very large, Cohen's *d* = 1.32. The combination of these findings indicates a clinically meaningful difference between the preoperative and postoperative corneal astigmatism.

### 3.3. Centroid Values of Preoperative Astigmatism, Postoperative Corneal Astigmatism, and SIA

**Figure 2:**
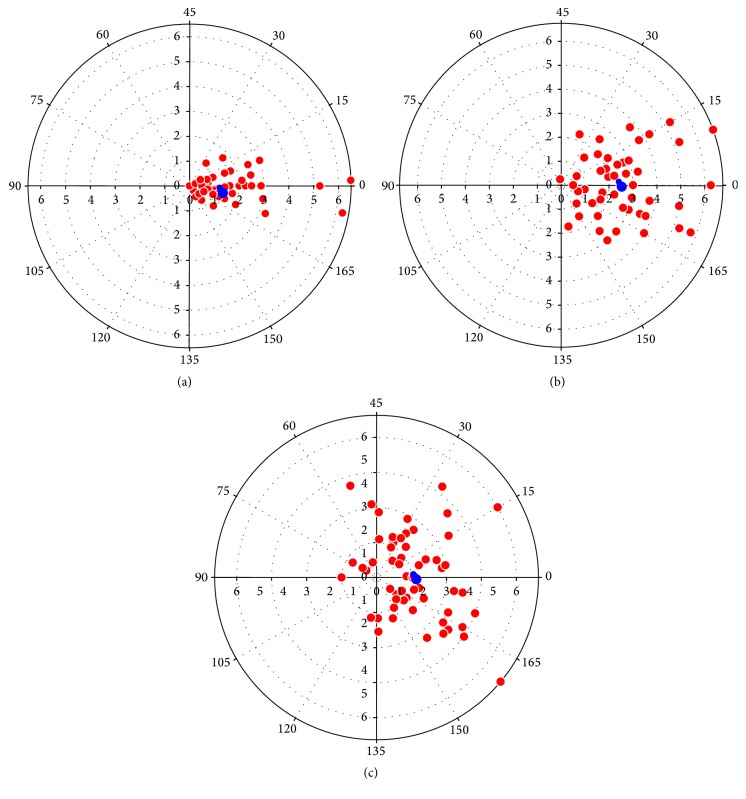
Double-angle plots of preoperative, 12-week postoperative, and surgically induced astigmatism. Coordinates of the preoperative astigmatism (a) are more clustered around its centroid value than the 12-week postoperative astigmatism (b) and the SIA (c). The coordinates are shown in red and the centroid value is shown in blue.

The results of the Cartesian coordinates based analysis are summarized in Table 2. The relatively high levels of coherence for the preoperative astigmatism, 12-week postoperative astigmatism, and SIA showed that the centroid values were reliable. The coherence level was the highest for the preoperative astigmatism (95%) followed by the 12-week postoperative astigmatism (89%) and lastly the SIA (66%). The coordinates of the preoperative astigmatism were more clustered around its centroid value than the 12-week postoperative astigmatism and the SIA. This is shown by the DAP in Figure 2.

The centroid preoperative astigmatism was 1.42 D × 179 (1.49 ± 1.34 D). This preoperative ATR astigmatism increased postoperatively at 12 weeks to 2.48 D × 0 (2.80 ± 1.40 D). The resultant centroid SIA was then recorded as 1.07 D × 1 (1.62 ± 0.90 D) showing ATR astigmatism.

## 4. Discussion

Placing incisions on the steeper corneal meridian has been recommended during MSICS with the idea that there is flattening of the meridian on which the incision is placed [16, 17]. Hence, with an on-axis incision, there is a reduction in the corneal power of the steeper meridian because of the flattening effect of the incision leading to minimal postoperative corneal astigmatism. For patients with ATR astigmatism who have a flatter vertical corneal meridian, it would be expected that a superior approach MSICS would flatten the already flatter vertical meridian leading to high postoperative corneal astigmatism. With senile cataract being the most common type of cataract in developing countries [19] and since there is an ATR shift in astigmatism with age [18], most cataract patients in developing countries may have preoperative ATR astigmatism. The choice of the location of incision for these groups of patients is therefore very important as that can influence the amount of postoperative corneal astigmatism.

Our results showed that the postoperative corneal astigmatism was significantly greater than the preoperative astigmatism (Figure 1). Thus, there was a significant increase in corneal astigmatism postoperatively. This increase in corneal astigmatism postoperatively was clinically meaningful (Cohen's *d* = 1.32). Previous studies have reported an ATR shift in astigmatism for patients who underwent superior approach MSICS [12, 16, 27–29]. Gokhale and Sawhney [28] reported centroid preoperative and postoperative astigmatism values of 0.18 × 90 and 1.10 × 3.3, respectively, for a group of cataract patients who underwent superior approach MSICS. Our reported centroid preoperative and postoperative astigmatism values were 1.42 D × 179 and 2.48 D × 0, respectively. Our centroid preoperative and postoperative corneal astigmatism were greater than those of Gokhale and Sawhney [28] (Table 2). Even though our reported preoperative and postoperative astigmatism were higher than those reported by Gokhale and Sawhney [28], both studies showed an ATR shift in astigmatism following superior approach MSICS. Thus, our results are consistent with the idea of an ATR shift in astigmatism following superior approach MSICS [12, 16, 27–29] as well as the expected increase in corneal astigmatism postoperatively following superior approach MSICS for this group of patients. This is mainly because of the flattening of the already flatter vertical meridian in ATR eyes.

Mallik et al. [12] reported a mean SIA value of 0.75 ± 0.4067 D for cataract patients with preoperative ATR astigmatism. This cohort of cataract patients however underwent temporal approach MSICS. Our reported SIA value for a similar cohort who underwent superior approach MSICS was 1.62 ± 0.90 D. Our reported SIA value was more than twice that reported by Mallik et al. [12]. Thus, our results are consistent with the idea that superior approach MSICS induces higher SIA than a temporal approach MSICS.

DAP of preoperative astigmatism, postoperative astigmatism, and SIA (Figure 2) show clustering of coordinates around the centroid value. This shows a good predictive value of the centroids obtained. Thus, it indicates that a superior approach will consistently induce centroid postoperative and SIA values of 2.48 × 0 and 1.07 × 1, respectively, for our cohort of cataract patients with preoperative ATR astigmatism. Good reliable values were also shown by DAP for superior and temporal approaches in a study done by Gokhale and Sawhney [28].

A superior approach may come with its own advantages and hence the reason why it might be more preferred by the less experienced surgeon over a temporal approach [30]. It does not require the surgeon to adapt to a different surgical position while a temporal approach does. It provides a forehead support for the surgeon's hands while a temporal approach does not as well as a difficulty in converting a temporal approach to a manual expression extracapsular cataract extraction (ECCE) technique. The above reasons may posit the preference of the superior approach over the temporal approach for the less skilled or inexperienced surgeon. However, since the postoperative corneal astigmatism is statistically and clinically significantly greater than the preoperative corneal astigmatism for ATR eyes when they undergo superiorly placed MSICS, a change in site of incision for this group of patients may be imperative. With senile cataract being the most common type of cataract in developing countries [19] and since there is an ATR shift in astigmatism with age [18], most cataract patients in developing countries may have preoperative ATR astigmatism. Hence, the ability of the surgeon to adapt to a change in site of incision for this group of patients is imperative even if that requires an emphasis on this approach during the training of these surgeons in the teaching hospitals in these developing countries. The high postoperative corneal astigmatism may create blurred images through a bigger circle of the least confusion on the retina. It may also produce glare and monocular diplopia.

## 5. Conclusions

We found a statistical and clinical significantly greater postoperative corneal astigmatism than preoperative corneal astigmatism for a group of ATR cataract patients who underwent superior approach MSICS. The high postoperative corneal astigmatism may create blurred images through a bigger circle of the least confusion on the retina. Since most cataract patients in developing countries may have preoperative ATR astigmatism, the ability of surgeons in these countries to adapt to a change in site of incision may be imperative.

## Figures and Tables

**Table 1:** Distribution of age.

Age group (years)	*n* (%)
30–39	2 (3.45)
40–49	3 (5.17)
50–59	5 (8.62)
60–69	19 (32.76)
70–79	25 (43.10)
80–89	4 (6.90)
Total	58 (100)

*n*: number of subjects in an age group.

**Table 2:** Results of the Cartesian coordinates based analysis.

Type of astigmatism	Mean ± SD		Centroid (mean ± SD)	Coherence (%)
	*X* value	*Y* value		
Preoperative astigmatism	1.42 ± 1.34	−0.03 ± 0.45	1.42 × 179 (1.49 ± 1.34)	95
12-week postoperative astigmatism	2.48 ± 1.42	0.03 ± 1.28	2.48 × 0 (2.80 ± 1.40)	89
SIA at 12 weeks	1.07 ± 0.97	0.05 ± 1.18	1.07 × 1 (1.62 ± 0.90)	66

SIA: surgically induced astigmatism; SD: standard deviation. Centroid values are presented as plus cylinders in diopters.
